# Comparison of Human Oocyte Activation Between Round-Headed Sperm Injection Followed by Calcium Ionophore Treatment and Normal Sperm Injection in a Patient With Globozoospermia

**DOI:** 10.3389/fendo.2020.00183

**Published:** 2020-04-07

**Authors:** Xiangli Niu, Qiuyan Ruan, Craig A. Witz, Weihua Wang

**Affiliations:** ^1^Research Center for Reproductive Medicine, Reproductive Hospital of Guangxi Zhuang Autonomous Region, Nanning, China; ^2^Houston Fertility Institute, Houston, TX, United States; ^3^Prelude-Houston Fertility Laboratory, Houston, TX, United States

**Keywords:** calcium ionophore, globozoospermia, artificial oocyte activation, human, oocyte

## Abstract

Fertilization failure is common in patients with round-headed sperm, a form of globozoospermia. Artificial oocyte activation is able to assist oocyte fertilization after sperm injection in these patients. Comparisons between oocyte fertilization with or without calcium ionophore have been reported in patients with round-headed sperm. However, no comparison has been reported between round-headed sperm injection followed by calcium ionophone activation and normal sperm injection. In this case report, half of oocytes from a patient were injected with her partner’s round-headed sperm followed by calcium ionophore activation, and the other half of oocytes were injected with a donor sperm without calcium ionophore activation. The injected oocytes were cultured to examine fertilization, embryo development, and embryonic aneuploidies in the resulting blastocysts. The fertilization rate was lower in round-headed sperm injected oocytes (3/6) than that in donor sperm injected oocytes (5/6), but rates of blastocyst and aneuploidies were similar in the resulting embryos between the two groups. A euploid blastocyst resulted from round-headed sperm injection was transferred, and a healthy baby was delivered. These results indicate that calcium ionophore treatment can assist oocyte activation in patients with round-headed sperm, but its efficiency to activate oocytes is lower than that induced by a normal sperm injection. However, embryo development and chromosome integrity may not be affected by calcium ionophore treatment.

## Introduction

Fertilization failure has been reported to occur in 1–5% of human *in vitro* fertilization (IVF) cycles after intracytoplasmic sperm injection (ICSI) (BR1). Most of these cases resulted from poor sperm morphology, such as globozoospermia (BR2–BR5). Globozoospermia is a rare form of teratozoopermia, mainly characterized by round-headed sperm that do not have or have very low level of phospholipase C-ζ (PLCζ), an important physiological agent inside sperm needed to induce oocyte activation (BR6, BR7).

Artificial oocyte activation (AOA) is a method of mimicking the physiological cell signaling mechanisms to activate oocytes, and calcium ionophore A23187 is the most widely used AOA method in humans (BR2–BR4, BR8–BR12). Deliveries of healthy babies have been reported since AOA by calcium ionophore was adopted (BR2–BR4, BR9–BR12). A systematic review of published reports demonstrated acceptable outcomes of calcium ionophore use, particularly for couples in which ICSI alone yielded poor or no fertilization (BR11). Recently, it has been reported that obstetric and neonatal malformations were not increased after ICSI and AOA (BR13).

Although oocyte activation was compared between AOA and non-AOA in patients with poor sperm morphology including round-headed sperm (BR5, BR14), comparisons have not been reported between round-headed sperm injection followed by AOA and normal (e.g., sperm from donor) sperm injection in the same cohort of oocytes or in the same patient. Aneuploidy formation in the resulting embryos between these treatments has not been compared either.

In this case report, a patient with a previously failed IVF cycle decided to use her partner’s round-headed sperm and sperm from a donor to inseminate her oocytes in an IVF cycle, and the oocytes inseminated with round-headed sperm were further processed for AOA. All resulting blastocysts were biopsied for examination of chromosome integrity (aneuploidy screening). The results were finally compared between two sperm samples.

## Materials and Methods

### Preparation of Calcium Ionophore

Calcium ionophore A23187 (Sigma-Aldrich, St. Louis, MO, United States) was used in the present case and 1 mg calcium ionophore (MW: 542.65) powder was dissolved in 922 μl dimethyl sulfoxide (Sigma-Aldrich) to make a 2 mM stock solution. The stock solution was aliquoted to 20 μl per tube and then stored at −20°C refrigerator until use.

Four hours before oocyte activation, one tube of calcium ionophore stock solution was warmed at room temperature and 5 μl of stock solution was added to 995 μl Gobal^TM^Total medium (LifeGlobal Group, CT, United States) to make a final concentration of 10 μM calcium ionophore working solution. This concentration was same as that in some commercial products (Kitazato, Japan and Gynemed, Germany). Micro drops (∼30 μl) were made with the working solution in a culture dish and the drops were covered with culture oil. A washing dish with six 50 μl drops of Gobal^TM^Total medium was also prepared for oocyte washing after treatment. Both dishes were placed in a CO_2_ incubator until use.

### Oocytes Retrieval and Sperm Preparation

The female patient underwent controlled ovarian stimulation for 11 days with a combination of daily injection of 300 IU recombinant follicle-stimulating hormone (Gonal-F, EMD Serono, MA, United States) and 300 IU of a combination of follicle stimulating hormone and luteinizing hormone (Menopur, Ferring Pharmaceuticals, NJ, United States). When the lead follicle reached 13 mm, 0.25 mg gonadotropin releasing hormone antagonist (Cetrotide, EMD Serono) was given daily until triggering for oocyte maturation by recombinant human chorionic gonadotropin (hCG) (Ovidrel, EMD Serono). When 3 follicles reached 17 mm or larger, 500 μg hCG was injected to induce final oocyte maturation that was Day 12 after stimulation. Oocytes were retrieved at 36 h after hCG administration and cultured in Global^TM^Total medium at 37°C in an atmosphere of 5.5% CO_2_, 6% O_2_, and balanced N_2_ under humidified conditions.

Fresh ejaculated semen was washed using Sperm Washing Medium (Fujifilm-Irvine Scientific, Irvine, CA, United States) to remove the seminal plasma. Frozen donor sperm were purchased from a commercial sperm bank, thawed and washed with Sperm Washing Medium also. Swim-up was used to collect motile sperm in both samples.

### ICSI and Calcium Ionophore Treatment

Cumulus cells were removed by using hyaluronidase (Fujifilm-Irvine Scientific) at 4 h after oocyte retrieval and metaphase II oocytes were injected 5 h after retrieval. The oocytes inseminated with partner’s sperm were activated with 10 μmol calcium ionophore in Global^TM^Total medium for 15 min immediately after ICSI, and the time was the same as that used by other report (BR14). This time is also suggested by commercial companies that provide the same product. After activation, oocytes were washed completely and then transferred to Global^TM^Total medium until fertilization assessment. Oocytes inseminated with donor sperm were cultured in Global^TM^Total medium without artificial activation until fertilization assessment.

### Assessment of Fertilization, Embryo Quality, and Blastocyst Biopsy

Fertilization was assessed 18 h after ICSI, and normal fertilization was characterized by two distinct pronuclei and two polar bodies. Embryo quality was evaluated by an inverted microscope on days 3, 5, and 6. Blastocysts at days 5 and 6 were biopsied using a 20 μm polished biopsy pipette (Sunlight Medical, Jacksonville, FL, United States) with assisted cutting by the ZILOS-tk laser system (Hamilton Thorn Bioscience Inc., MA, United States). The biopsied cells were collected and screened to exam chromosomes of the embryos. All blastocysts were cryopreserved for later frozen embryo transfer (FET).

### Chromosome Analysis in the Blastocysts

Biopsied samples were analyzed for preimplantation genetic testing for aneuploidies (PGT-A) by a commercial genetic testing company (Invitea, San Francisco, CA, United States) using Illumina platform with a FAST-SeqS next generation of sequencing method and associated bioinformatics pipeline validated for accurate detection of whole chromosome number, segmental (≥10 Mb) aneuploidy, polyploidy, and UPiD (chromosomes 1–16. 18, and X). In the report, normal or euploid embryos is defined as a test result indicating that embryo has the correct number of all chromosomes and does not have ≥10 Mb of duplication and/or deletion segment. All procedures were in accordance to the guidelines for good practice developed by the Preimplantation Genetic Diagnosis International Society (BR15).

### Blastocyst Vitrification, Warming, and Transfer

The biopsied blastocysts were vitrified using the Cryotop device and vitrification kit (Fujifilm-Irvine Scientific). Both equilibration solution and vitrification solution were warmed in original vials at 37°C for at least 30 min before use. Briefly, collapsed blastocysts by a laser pulse were equilibrated in 100 μl drop (without oil cover) of equilibration solution for 2 min, and then 45 s in 100 μl drop (without oil cover) of vitrification solution (both steps were performed on a 37°C warming stage) (BR16, BR17) before loading to Cryotops. All blastocysts were vitrified individually and then stored in liquid nitrogen until warming for FET.

For warming, blastocyst was exposed to a thawing solution (Fujifilm-Irvine warming kit) at 37°C for 1 min, transferred to a dilution solution for 3 min and finally to a washing solution for 10 min with a solution change after 5 min at room temperature. After completion of the warming process, blastocyst was cultured in Global^TM^Total medium for 2 h before transfer.

For preparation of the transfer, the patient received 2 mg estradiol tablets (Estrace, Warner Chilcott, NJ, United States) orally, three times a day and 0.1 mg, every 3 days, estradiol patch (Estradiol Transdermal System, Noven Pharmaceuticals, NJ, United States). Intramuscular administration of 100 mg/daily progesterone (Progesterone Oil, West-Ward Pharmaceuticals, NJ, United States) was initiated after 17 days of estradiol treatment. The blastocyst was transferred on the sixth day of progesterone administration and progesterone was continued daily until the first serum β-hCG test 2 weeks after transfer. Ongoing pregnancy was supported by continued estradiol and progesterone until 11 weeks of pregnancy. Pregnancy was initially confirmed 14 days after embryo transfer by a serum β-hCG assay. Four weeks after embryo transfer, when a gestational sac and a heartbeat appeared, the patient was diagnosed as having a clinical pregnancy. The patient was then monitored by an obstetrician until childbirth.

### Preliminary Test for Oocyte Activation With Calcium Ionophore

A preliminary test was conducted to examine the efficiency of oocyte activation with calcium ionophore before this case started. Donated frozen oocytes were activated with 10 μmol calcium ionophore in Global^TM^Total for 15 min without sperm injection. After activation, oocytes were washed and cultured in Global^TM^Total medium until assessment of pronuclear formation after 18 h of culture and subsequent embryo development by day 6.

## Results

### Preliminary Oocyte Activation Efficiency With Calcium Ionophone

In the preliminary test, 20 warmed metaphase II oocytes were activated with calcium ionophore and 12 (60%) oocytes were activated to release the second polar body and form one pronucleus, indicating that oocytes underwent the second meiosis normally after artificial activation with ionophore. When the activated oocytes were further cultured, it was found that all activated oocytes develop to 6–8 cells by day 3; however, no embryo developed to blastocyst by day 6.

### Patient Infertility History

A 37 year old woman and her 36 year old husband presented with primary male infertility for 8 years. The couple had a previously failed IVF cycle in other IVF clinic 6 years ago. It was reported that 25 oocytes were collected and 7 oocytes were fertilized. The semen analysis for that IVF cycle indicated that 80% of sperm were round-headed sperm. Embryo transfer was not performed due to poor embryo development. There was no recorded infertility treatment until the couple visited our clinic. The husband had globozoospermia with 100% round-headed sperm in two semen analyses including stained sperm morphology assessment (total motile sperm were 73.5 million and 20.9 million, respectively) in 2016 and 2018. The last semen analysis in 2018 indicated that 2.3 ml semen was collected. Sperm concentration was 51 × 10^6^ sperm/ml and motility was 17.8%, resulting in a total of 20.9 × 10^6^ motile sperm. However, no sperm had normal morphology in the sample and all were round-headed sperm. Due to previous poor fertilization and lack of embryo development, the couple decided to inseminate half of oocytes with the husband’s sperm followed by AOA with calcium ionophore, and the other half of oocytes with donor sperm without AOA in an IVF cycle planned in 2018.

### Case Presentation

**FIGURE 1:**
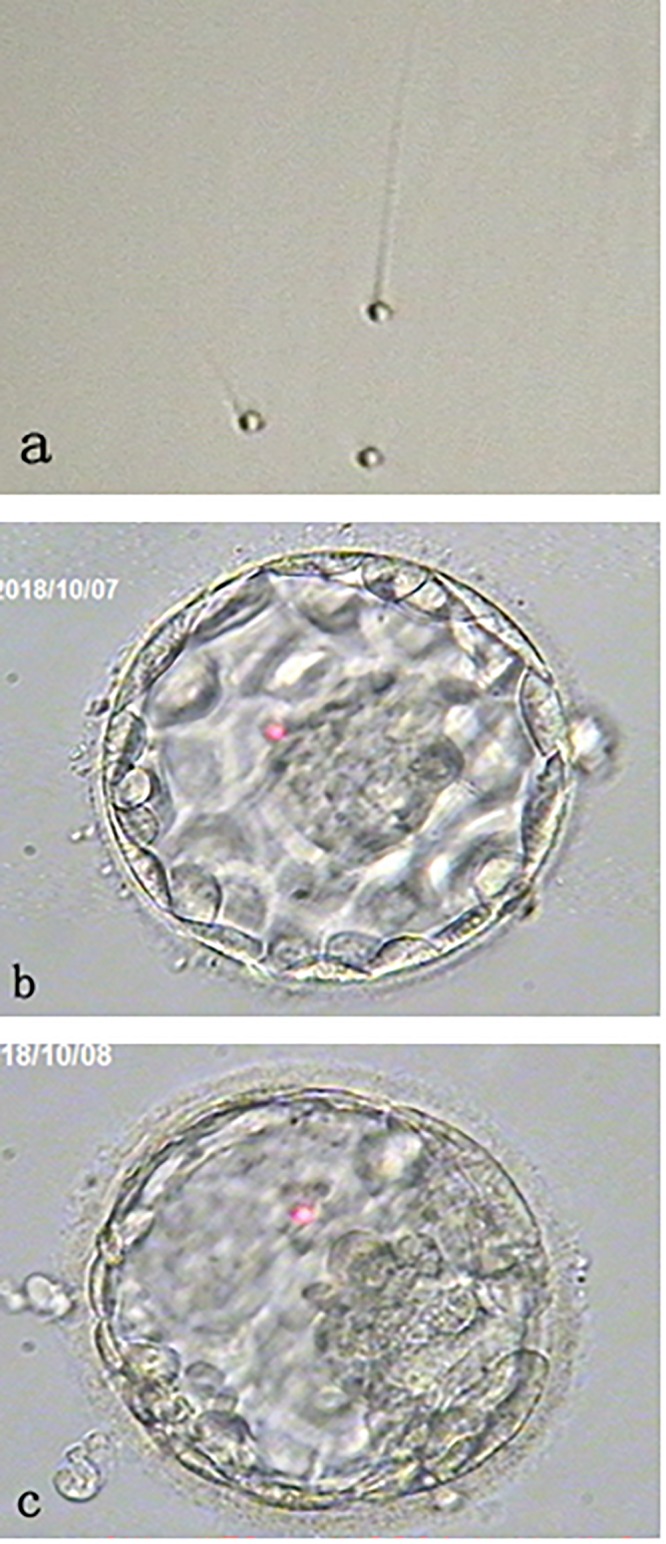
Morphology of round-headed sperm and the blastocysts. **(a)** shows small round-headed sperm. **(b)** shows a Day 5 good blastocyst with normal chromosome number (46, XX); while **(c)** shows a Day 6 aneuploid blastocyst. Both blastocysts were resulting from the oocytes activated by calcium ionophore after round-headed sperm injection.

Patient’s fresh sperm was collected at the oocyte retrieval day and semen analysis revealed sperm counts of 84 × 10^6^/ml with 45% motile and 100% round-headed sperm under × 400 magnifications without sperm morphology staining (Figure 1a).

Thirteen oocytes were retrieved and 12 were found to be metaphase II. Metaphase II oocytes were divided into two groups and each 6 oocytes were inseminated with either patient’s sperm or donor sperm. It was found that 3 out of 6 oocytes injected with round-headed sperm and activated by ionophore fertilized to form 2 pronuclei. All fertilized oocytes cleaved at Day 3 examination, and 2 developed to blastocysts (Figures 1b,c). By contrast, 5 out of 6 oocytes injected with donor sperm fertilized normally, all cleaved at Day 3 examination, and 3 developed to blastocysts.

All blastocysts were biopsied for aneuploidy screening with next generation sequencing. As results, one euploid (46, XX) blastocyst with good inner cell mass (ICM)/good trophectoderm (TE) (Figure 1b) and one aneuploid blastocyst [45, X, dup(Y) (p11.2p11.2), del (1) (q31)] with fair ICM/good TE (Figure 1c) were obtained from the oocytes injected with round-headed sperm and activated by ionophore. By contrast, 2 euploid (46, XX) blastocysts (one with good ICM/good TE and the other with fair ICM/good TE) and 1 aneuploid (45, XY, −22) blastocyst with fair ICM/fair TE were obtained from the oocytes injected with donor sperm. Blastocyst morphology classification was based on the standard developed by the Society for Assisted Reproductive Technology (BR18).

After transfer of one euploid blastocyst resulting from the patient’s round-headed sperm and AOA, the blastocyst implanted and a healthy girl (weight 7.13 lb) was delivered at gestation of 40 weeks and 5 days by cesarean section. No neonatal care was recorded and reported.

## Discussion

The present case report is the first to compare fertilization, embryo development, and chromosomal integrity between round-headed sperm and normal sperm in the same cohort of oocytes from the same patient. In this report, some new information has been added to the current understanding of human AOA.

First, although AOA can improve fertilization in patients with round-headed sperm as compared with the oocytes without AOA (BR5, BR14), the fertilization rate appears to be lower than a normal sperm injection without AOA (current case). The overall fertilization rates from previous reports were less than 50% (BR2, BR14) with 100% round-headed sperm. However, the fertilization rate may be higher if samples had partial round-headed sperm (BR4, BR14). It is proposed that oocyte activation injected with round-headed sperm may mainly rely on AOA. We obtained 60% of oocyte activation rate in the preliminary test in which frozen/warmed oocytes were treated with AOA without sperm injection and 3 out of 6 oocytes fertilized in round-headed sperm-injected oocytes followed by AOA, which was lower than that in donor sperm injected oocytes (5 out of 6). Lower activation rates of oocytes with calcium ionophore were reported not only in human, but also in other mammals (BR19–BR22). The explanation for this is that normal sperm fertilization can induce multiple calcium oscillations (BR23), while calcium ionophore can induce only one calcium oscillation (BR22) and one calcium oscillation may be not enough to initialize oocyte activation events in some oocytes. Furthermore, oocyte activation is also affected by other factors, such as oocyte maturation status (BR10, BR24–BR26). It has been found that aged oocytes are more easily activated by AOA, such as failed fertilized oocytes after ICSI or purposely aged oocytes (BR10, BR25, BR26).

Another reason for the lower fertilization rate may be due to different levels of PLCζ in round-headed sperm, which may have co-effects on activation in human oocytes. For example, if some level (more or less) of PLCζ is present in the round-headed sperm, it may have a synergetic effect with AOA on oocyte activation, while if no PLCζ is present in the sperm, AOA alone may have a limited effect to induce oocyte activation. This may explain why lower fertilization rates were obtained with 100% round-headed sperm than that with partial round-headed sperm or normal sperm [2, 4, current case]. However, such a hypothesis was not examined in the present case report and it requires further investigation.

Second, once the oocytes are activated, blastocyst development may be similar between round-headed sperm injection followed by AOA and normal sperm injection without AOA. For example, in this report, 2 out of 3 oocytes fertilized by round-headed sperm and AOA developed to blastocysts, while 3 out of 5 oocytes fertilized by donor sperm developed to blastocysts. These results were similar as those in the previous case reports, showing that ∼50% of fertilized oocytes developed to blastocysts (BR2, BR4). Although the numbers of oocytes in this case were limited, these results may indicate that the activated oocytes have a similar developmental potential between round-headed sperm and normal sperm. However, lower blastocyst development was reported in a previous study with ICSI and AOA (BR14). The reason(s) for such differences observed between case reports is complicate as most of these reports have limited cycle number or oocyte number.

Third, it would appear that chromosomal integrity was not affected by AOA in the oocytes injected with round-headed sperm. We observed that human oocytes activated with ionophore have normal meiosis by releasing the second polar body and forming one pronucleus in our preliminary experiment. This is important for subsequent mitosis because diploid embryo need to be produced after sperm injection. If oocytes do not release the second polar body, or form two female pronuclei, then a triploid embryo would be produced after sperm injection. Such a situation was reported in other animal (BR19) but appears to not be a case in human oocytes. In the present report, aneuploid (not triploid) blastocysts were observed in both groups, which may originate from oocytes, not from treatments, as quite high rates of embryonic aneuploidy were observed in human IVF (BR26). Furthermore, it has been suggested that sperm DNA fragmentation may be high in globozoospermia (BR5). More aneuploid embryos may be created if the sperm with fragmented DNA are used for ICSI (BR27, BR28). However, this was not examined in the present case report.

It has been found that embryo implantation and live birth rates were very low in patients with AOA after ICSI (BR1, BR11, BR13, BR14) and many pregnancies were lost during the first trimester (BR1) although it was not known if this was resulting from poor sperm quality and/or poor oocyte activation. Aneuploidy is high in human embryos produced by IVF, even in young women (BR29), suggesting that low implantation rates and early pregnancy lose reported in the previous studies may be partially due to embryonic aneuploidies originated from oocytes (BR29) and/or sperm (BR5). Therefore, screening of the resulting embryos may be necessary for selection of euploid embryos to transfer, especially in patients with advanced maternal ages and poor sperm morphology, so that the overall pregnant rate can be increased.

Although the use of calcium ionophore treatment after ICSI has been shown to improve fertilization as well as overall pregnancy and live birth rates, no information on the long term health of the offspring is yet available except a recent study in which 47 children have been followed (BR13). Because calcium oscillatory patterns in the fertilized oocytes may affect gene expression and development to term in mouse (BR30), it is necessary to further reveal the detailed events within human oocytes after ICSI and AOA.

Previous study found that ionophore works for some patients, but does not work for others (BR1, BR14). So far, we have had only one case in our clinic, hence we still do not know what caused the different outcomes between patients, treatments or clinics. Globozoospermia is responsible for less than 0.1% of male infertility (BR5), thus sharing of success of various treatments is important for human IVF so that clinics can chose the best available treatment option if they have such cases.

Some other reagents and/or methods have been applied to achieve AOA, such as electric current (BR31, BR32), ethanol (BR33), strontium chloride (BR34), and puromycin (BR8). There are still very limited reports on comparison of different activation reagents on human oocyte activation and embryo development (BR8, BR31, BR34) but it appears that calcium ionophore has better efficiency than strontium chloride (BR34). Electric current can also activate human oocytes (BR31, BR32) but it has not been applied in human IVF due to expensive cost of equipment. Calcium ionophore A23187 is an easy to use product so it has been widely used in human AOA, which has also resulted in development of commercial oocyte activation products containing calcium ionophore (Kitazato, Japan and Gynemed, Germany). Some reports indicated that repeated (double) treatments of oocyte with calcium ionophore (BR14) or combined treatment of oocytes with calcium ionophore and other AOA (BR8, BR31) can increase the fertilization rates. However, there is still no success case report being published with these approaches.

Globozoospermia is most commonly caused by mutations in the *DPY19L2* gene (BR35). Mutations in other genes likely also cause globozoospermia (BR35–BR37). In the present case, we noticed that the percentage of round-headed sperm changed in this patient from 80 to 100% during the 6-year interval. Given the genetic nature of globozoospermia, it is very unlikely that a patient will progress from partial to complete globozoospermia. The semen analysis were performed by two different laboratories, so we do not know what caused the different proportions of round-headed sperm in the samples. It might be related to the sperm assessment technique. However, the most recent three semen samples were analyzed in our laboratory by two andrologists (semen analysis) and two embryologists (semen processing for ICSI), and it was confirmed that 100% of typical round-headed sperm (Figure 1a) were found in the samples and round-headed sperm were used for ICSI.

In conclusion, the present report indicates that AOA with calcium ionophore can induce normal oocyte meiosis resumption, which is important for producing diploid embryos after sperm injection. This activation method is beneficial to globozoospermia patients by rescuing oocyte fertilization and embryo development. Although fertilization can be improved in these patients by using AOA, the fertilization rate is still lower than that of normal sperm injection. The current AOA method may not be the most optimal. Furthermore, our data indicate that once the oocytes are activated and fertilized normally, subsequent embryo development and chromosome integrity (ploidy status) may not be affected by AOA in human oocytes. Further study is necessary to address other genetic or epigenetic differences between the embryos or individuals resulting from AOA and normal fertilization.

## Data Availability Statement

The data that support the findings of this case report are available on request from the corresponding author.

## Ethics Statement

The patients signed the consents for all laboratory and clinical procedures including controlled ovarian stimulation, fertilization of oocyte with partner’s sperm and donated sperm, embryo cryopreservation, oocyte activation with ionophore and chromosome assessment or PGT-A. The data was collected from medical records at the clinic and laboratory, and the study with IVF and PGT-A was approved by the New England Institutional Review Board (NEIRB 14-504). Written informed consent was obtained from the patient for the publication of any potential identifiable images or data included in this article.

## Author Contributions

CW managed the patient. XN, QR, and WW wrote the manuscript. All authors approved the final manuscript.

## Conflict of Interest

The authors declare that the research was conducted in the absence of any commercial or financial relationships that could be construed as a potential conflict of interest.

## Abbreviations

AOA: artificial oocyte activation
FET: frozen embryo transfer
hCG: human chorionic gonadotropin
ICM: inner cell mass
ICSI: intracytoplasmic sperm injection
IVF: *in vitro* fertilization
PGT-A: preimplantation genetic testing for aneuploidies
PLC ζ: phospholipase C- ζ
TE: trophectoderm.
